# Spatio-Temporal Evolution and Driving Factors of Rural Settlements in Low Hilly Region—A Case Study of 17 Cities in Hubei Province, China

**DOI:** 10.3390/ijerph18052387

**Published:** 2021-03-01

**Authors:** Shukui Tan, Maomao Zhang, Ao Wang, Qianlin Ni

**Affiliations:** 1 College of Public Administration, Huazhong University of Science and Technology, Wuhan 430079, China; tansk@hust.edu.cn (S.T.); lideshou@mails.ccnu.edu.cn (Q.N.); 2 School of Civil Engineering, University of South China, Hengyang 421001, China; wennie@mails.ccnu.edu.cn

**Keywords:** rural settlements, spatio-temporal evolution, driving forces, geographic detectors, geographically temporally weighted regression model (GTWR), Hubei Province

## Abstract

With the rapid development of the social economy, factors of social and economic development in China’s rural areas have been continuously reorganized, and the pattern and distribution of rural residential areas have undergone significant changes. In rural areas, there have been many peculiar phenomena of “reducing people but not reducing land in rural areas, which has caused tremendous pressure on land resource protection. We used geographic detectors and a geographically temporally weighted regression model (GTWR) to explore the rural settlements’ evolution and driving mechanism in Hubei Province from 1990 to 2015. The results show that the kernel density of rural settlements decreased from 1.62 villages/km^2^ in 1990 to 1.60 villages/km^2^ in 2015. The scale of rural residential patches has obvious regional differentiation characteristics. From southeast to northwest, there is a wave-like distribution structure of “high-low-high-low-high”, and the clustering characteristics of “cold and hot spots” are strengthened with time. Based on GTWR analysis, the total rural population, total power of agricultural machinery, and rural electricity consumption have promoted the expansion of rural settlements, with the regression coefficients 0.096, 0.484, and 0.878, respectively. Cultivated land, agricultural output value, and rural labor force have negative impacts on the expansion, the regression coefficients of the village were −0.584, −0.510, and −0.109, respectively.

## 1. Introduction

As an external manifestation of the rural economy and society, rural settlements are the basic spatial unit of the rural population’s production and life [1,2], and one of the types of land use that significantly reflects the relationship between man and land [3,4]. Rural settlements not only carry all aspects of rural production and life but also play an important role in the direction and mode of rural sustainable development [5]. With the rapid development of the social economy and the acceleration of urbanization, the restructuring of rural areas has accelerated, the elements of rural social and economic development have been continuously reorganized, and the morphology and distribution of rural residential areas have undergone significant changes [6,7,8]. According to the latest global demographic analysis, there is about 45% of rural population in the world, but it is estimated that the rural population will only account for 30% in 2050 [9]. Rapid urbanization has accelerated the conversion of rural residential land to urban construction land [10]. This excessively rapid development process not only destroys the ecological environment, but also brings tremendous pressure to the life and production of rural residential areas [11,12,13].

So far, more than half of China’s people live in villages, and rural settlements are the main form of the rural population in China [14,15]. Since China’s reform and opening up in 1978, rapid economic growth and urbanization have created a continuous and urgent demand for construction lands [16,17]. The expansion of construction land, especially the expansion of urban land space, has become the most important feature of my country’s current land-use changes, while rural residential land has shown a slow-growth trend under the dual pressure of its own economic development and urban land expansion [18]. However, the rural population continued to decrease during the same period, and the slow increase in the scale of land use means a rapid increase in land use per capita. Land use in rural residential areas has become increasingly extensive, and there have been many strange phenomena of “reducing people without reducing land in rural areas”, which has caused tremendous pressure on the protection of cultivated land resources. There are still many problems in the development of such cities and rural areas. When the population continues to flow into the cities, can we withstand a series of urban diseases and shortages of cultivated land brought about by the development of urbanization? Facing the various problems and pressures of urban and rural development, rational planning and construction of land for rural residential areas is essential. Research on the temporal and spatial characteristics of rural residential land and exploration of sustainable development can pave the way for the development of human life in the future, while also alleviating some of the problems caused by urbanization and reducing the pressure of cultivated land occupation [19]. Moreover, during the rapid evolution of the spatial and temporal patterns of rural residential areas, there have been phenomena such as disorderly expansion, hollowing out of villages, and low land-use efficiency. The geographical differentiation and pattern evolution of the location, scale, distribution, structure, form, and function of rural residential areas can often reveal the footprints of human-land interaction at different stages and regions. Therefore, exploring the temporal and spatial evolution of rural residential areas and the mechanism of influencing factors has important theoretical value and practical significance for formulating correct spatial development strategies [20], optimizing the spatial pattern of rural residential areas, and promoting sustainable rural development.

In recent years, many scholars have conducted in-depth research on the spatial distribution and evolution of rural residential areas from different perspectives, and have obtained rich research results. The distribution and evolution process of rural settlements are mostly affected by the natural environment, social economy, culture, and other factors, but their dominant factors have significant regional differences [21,22]. At the same time, their influence process and driving mechanism have also attracted much attention. A large number of scholars have used analytical methods such as the spatial rhythm index, geographic detectors, and spatial correlation measurement models to explore the changes in the scale, density, and shape of rural settlements within different elevations and slopes [23,24]. Some scholars also used the methods to analyze the driving mechanism of the ecological environment, natural disasters, ecological migration, and other sudden factors on the spatial evolution of rural residential areas [25,26].

The changes in the scale, form, and structure of rural settlements reflect the process of social and economic changes in the context of urbanization, industrialization, and agricultural modernization, as well as in-depth transformations such as changes in farmers’ livelihoods and their social roles [27,28,29]. This indicates that the current social-economic factors play a leading role in the spatial evolution of rural settlements. Therefore, many scholars have studied the driving forces of rural residential land-use change mainly focusing on the humanities, and the main factors include technological progress, population non-agriculturalization, income level, industrial structure transformation, rural function transformation, residents’ lifestyle, land system, etc. [30,31]. These studies explored the correlation between the increase in rural residential land area and the growth of rural population, township industrial output value, per capita GDP, and per capita net income of farmers. For example, Elvin E, Skinner G, etc. believed that before modern times, the natural environment played a decisive role in the location and scale of settlements, but with social development, the economic structure changed the settlement structure [32]. Robinson claimed that land management systems, family risk diffusion strategies, and regional factors have an impact on the rural settlement pattern in Cape Town, South Africa [33].

In the exploration of the influencing factors of rural residential areas, a large number of studies have used spatial econometric models, especially classical geographic weighted regression models have been applied widely [34,35]. Although classical geographically weighted regression can solve the problem of spatial heterogeneity that traditional linear regression models cannot deal with to a certain extent, it ignores the spatial heterogeneity of the time dimension and the scale difference, which is somewhat lower than the estimated value of panel data. Therefore, there is a certain estimation error in the analysis process of the classical geographic weighted regression model. Scale can be considered as the most important topic of geographic information science, and a fundamental element of geographical research [36]. The development process of different types of socio-economic factors often corresponds to different time and space scales. The emergence of a certain socio-economic phenomenon is often determined by multiple temporal and spatial processes of different scales. Huang et al. proposed a temporal and spatial geographic weighting (GTWR) model [37], which takes the changes in the time dimension into account and overcomes the shortcomings of the geographically weighted regression model. The model can use regional panel data to perform spatial regression, that is, to link the time attribute to the spatial attribute of the geographically weighted regression, to reflect the temporal and spatial change information of the study area better, and make the estimation result more effective.

To sum up, the scale, form, and spatial layout of rural settlements are the result of a series of factors such as natural and human factors. With the continuous progress of the social economy, the degree of restriction on rural settlements was gradually weakened by natural conditions. However, the social and economic conditions of human social and economic activities are the main factors affecting the changes in rural settlements. In view of this, this article is based on the six periods of land use data and socio-economic data in 1990, 1995, 2000, 2005, 2010, and 2015 of 17 prefecture-level cities in Hubei Province, China. The rural settlement’s spatial distribution, evolutionary characteristics, and the influencing mechanism of social-economic factors have been analyzed. The key advantages that may exist in this article are as follows: (1) Hubei Province is located in the middle and lower reaches of the Yangtze River, which is the earliest residential area of the Chinese nation. The current pattern of rural settlements is the result of long-term evolution, which helps to study its evolution process. (2) The socio-economic development levels of 17 cities in Hubei Province are significantly different. The socio-economic development levels of Wuhan, Xiangyang, and Yichang are expressively higher than other cities in Hubei. This is more diversified in the research on the distribution characteristics, evolution, and influencing factors of rural residential areas in this region, and can represent the evolution trend of rural settlements. (3) We use the GTWR model to comprehensively consider the spatio-temporal attributes of the data, which can help the research results to be more robust and accurate.

## 2. Materials and Methods

### 2.1. Study Area

**Figure 1:**
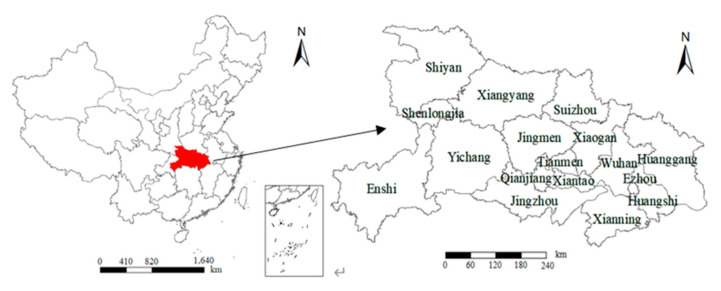
Administrative area map of Hubei Province.

Hubei Province is located in central China, with a geographical location ranging from 29°05′ N to 33°20′ N and 108°21′ E to 116°07′ E. The terrain is roughly surrounded by mountains on three sides in the east, west, and north, with a low level in the middle and an incomplete basin that is slightly open to the south. The terrain in the province is diverse, with mountainous hills and plains. The location of Hubei Province is shown in Figure 1. There is a famous typical lacustrine plain located here, which is called Jianghan Plain. Most of the ground is 20–100 m above sea level, the terrain is flat and the soil is fertile. Except for the high mountains, most of the area has a humid subtropical monsoon climate, with sufficient light energy, abundant heat, a long frost-free period, abundant precipitation, and rain and heat in the same season. The flat terrain, abundant water resources, and excellent climate have created a long history of farming and culture. Hubei Province is an important food production base in China with obvious advantages in agricultural and sideline product resources.

### 2.2. Data Sources

The vector data of rural settlements comes from the Resource and Environmental Science Data Center of the Chinese Academy of Sciences (http://www.resdc.cn). This paper used 6 Landsat TM/ETM remote sensing images from 1990, 1995, 2000, 2005, 2010, and 2015 as the data source, and applied human-computer interaction to interpret land use/land cover into cultivated land, forest land, and land cover. The total classification accuracy of grassland, construction land, water area, and unused land was over 94.31%. We extracted vector data of rural residential spots in construction land and generated a point file based on its geometric center for spatial analysis of residential spot density. Socio-economic development data came from the “China Regional Economic Statistical Yearbook”, “China Provincial (City) Social and Economic Statistical Yearbook” and the statistical yearbooks of various provinces. Among them, the total population, the area of arable land at the end of the year, the total agricultural output value, the total power of agricultural machinery, the electricity consumption in rural areas, and the use of chemical fertilizers were from the “Hubei Rural Statistical Yearbook”. The number of large livestock, total grain output, aquatic product output, and afforestation area data came from the “China County (City) Social and Economic Statistical Yearbook”.

### 2.3. Methods

#### 2.3.1. Kernel Density Estimation

Kernel density estimation was used to calculate the density of elements in their surrounding neighborhoods. It is a statistical non-parametric density estimation method. This method can intuitively analyze the degree of aggregation of the spatial distribution of rural residential areas. The specific principle is as follows: take each sample point (x,y) of rural residents to be observed as the center, and calculate the density contribution of each sample point within the specified radius (width h) of the center point of each grid unit through the kernel function value, search for the center point of the grid cell in the radius range. The closer to the sample point, the greater the density value. The mathematical model is as follows.

(1) $$f_{n}=\frac{1}{nh}\sum_{i=1}^{n}k\left(\frac{x-x_{i}}{h}\right)$$

In the formula, f(x,y) refers to the calculated value of the nuclear density of a certain rural residential spot location (x,y) in the research area, and n is the number of rural residential spot patches. h is the bandwidth, that is, the search radius distance, and k is the kernel density function. $d_{i}$ is the distance to be observed between the rural residential area location (x,y) and the rural residential area location $\left(x_{i},y_{i}\right)$ of the i-*th* sample. The higher the value, the greater the spatial distribution density of rural residential areas, and vice versa. This paper used ArcGIS 10.2 software to analyze the nuclear density results.

#### 2.3.2. Spatial Hot Spot Analysis

Spatial hot spot analysis, namely local clustering test $\left(Getis-OrdG_{i}^{*}\right)$, can be used to analyze whether there are obvious high-value clustering features and low-value clustering features in the scale of rural settlements in local areas [38,39]. The specific principle is as follows: by calculating the local sum of a certain rural residential element and its neighboring rural residential elements within a given distance, the calculation result is compared with the sum of all elements in the area and used to analyze the rural area. The degree of agglomeration of the attribute values of the residential elements at the local spatial level and the result can reflect the spatial differentiation of the scale of rural residential areas, that is, whether there are “cold spots” and “hot spots” areas in local vicinities. The mathematical model is as follows:

(2) $$G_{i}^{*}\left(d\right)=\sum_{j=1}^{n}w_{ij}\left(d\right)x_{j}/\sum_{j=1}^{n}x_{j}$$

where $G_{i}^{*}\left(d\right)$, refers to the $G^{*}$ value of the element i of rural residential area to be measured, n is the total number of rural residential area patches, $w_{ij}\left(d\right)$ is the spatial weight within the range of distance d, and $x_{j}$ is the j rural area property value of the residential area. If the distance between the rural residential area unit (j) and the rural residential area unit (i) to be measured is less than the critical distance d, the spatial weight matrix $w_{ij}\left(d\right)$ is 1, otherwise it is 0.

In order to facilitate an understanding of $G_{i}^{*}\left(d\right)$, the following formula can be used for standardization.

(3) $$Z\left(G_{i}^{*}\right)=\left[G_{i}^{*}-E\left(G_{i}^{*}\right)\right]\left[G_{i}^{*}-E\left(G_{i}^{*}\right)\right]/\sqrt{VAR\left(G_{i}^{*}\right)}$$

where $E\left(G_{i}^{*}\right)$ is the mathematical expected value of $G_{i}^{*}\left(d\right)$, and $VAR\left(G_{i}^{*}\right)$ is the mathematical variance of $G_{i}^{*}\left(d\right)$. If $Z\left(G_{i}^{*}\right)$ > 0, it means it belongs to the “hot spots”area with high-value clusters. Otherwise, it belongs to the “cold spots” area with low-value clusters.

#### 2.3.3. Rural Land Expansion Intensity Index

The rural land expansion intensity index represents the phase change rate of the rural residential land area of a spatial unit, and can directly reflect the change range and speed of the residential land area of each spatial unit [40]. Calculating the expansion intensity index of each residential area can study the development speed and status of the residential area. After standardization, the expansion speed of residential land at different stages can be comparable. The calculation formula is as follows.

(4) $$UER_{i}=\frac{U_{i}^{t2}-U_{i}^{t1}}{U_{i}^{t1}\times \Delta t}$$

In the formula: $UER_{i}$ represents the intensity index of rural residential land expansion, $U_{i}^{t1}$ and $U_{i}^{t2}$ are the rural residential land area of the spatial unit i during the study period $t_{1}$ and $t_{2}$, respectively, and t is the research time span.

#### 2.3.4. Geographic Detector Analysis

A geographic detector is a spatial analysis model for measuring the relationship between geographic phenomena and their potential influencing factors [41]. It provides a better expression of the similarities in the same area and the differences in different areas, meanwhile, it explains the explanatory strength of the independent variable X to the dependent variable Y. This paper applied geographic detectors to analyze the relationship between the potential influencing factors and the land area of rural residential areas, and then identify invalid factors and dominant factors. The calculation formula is as follows.

(5) $$q=1-\frac{1}{n\sigma^{2}}\sum_{h=1}^{L}n_{h}\sigma_{h}^{2}$$

In the formula: n is the total number of samples in the study area (pieces), $\sigma^{2}$ is the discrete variance of the Y value of the entire area, h is the partition of variable Y or factor X, h = 1, 2, …, L, L represents the number of partitions (pieces), q is the spatial heterogeneity of an indicator, and the range of values is [0, 1]. If the partition is generated by the independent variable X, the larger the value, the stronger the explanatory power of the independent variable X for the attribute Y.

#### 2.3.5. Geographically and Temporally Weighted Regression Model (GTWR)

The traditional regression model often ignores the spatial structure of the data when estimating regional data, and generally, it can only get the average estimated value. The Geographically Weighted Regression (GWR) model proposed by Fotheringham considers spatial non-stationarity [42]. The influence of explanatory variables varies from region to region, and it is an important tool for studying spatial heterogeneity. In actual application, the GWR model also has certain defects. For example, this model can only study cross-sectional data and lacks time dimension considerations. In the research, the situation at different time nodes could be studied to illustrate the problem, but compared with the panel data node data, it had a certain contingency and there were still some deviations in the estimation results. The temporal and spatial geographic weighting (GTWR) model proposed by Huang et al., which comprehensively considers the changes in the time dimension, overcomes the shortcomings of the geographic weighted regression model [37]. The model could use regional panel data to perform spatial regression, that is, to link the time attribute to the spatial attribute of the geographically weighted regression, to reflect the temporal and spatial change information of the study area better, and make the estimation results more effective. This article introduced the GTWR model to solve the problem of spatial heterogeneity. The expression formula is as follows.

(6) $$Y_{i}=\beta_{0}\left(u_{i},v_{i},t_{i}\right)+\sum_{k=1}^{p}\beta_{k}\left(u_{i},v_{i},t_{i}\right)X_{ik}+\varepsilon_{i}$$

In the formula: Y and X are the explained variables and explanatory variables respectively, i is the sample area, u and v are the coordinates of the sample area, t is the time, $\beta_{0}\left(u_{i},v_{i},t_{i}\right)$ is the intercept term, $\beta_{k}\left(u_{i},v_{i},t_{i}\right)$ is the estimated coefficient of the explanatory variable, β > 0 means that the explanatory variable is positively correlated with the explained variable, and $\varepsilon_{i}$ is the random disturbance term.

The core component of GTWR is the selection of the spatial weight function, and the spatial correlation of data is realized by the construction of a spatial weight matrix. In this paper, the spatial-temporal geographic weighted regression model used the Gaussian function method proposed by Huang et al. [37], the spatial-temporal weight function, and spatial-temporal distance, which combined the two-dimensional information of the temporal and spatial dimensions effectively. The calculation formulas are as follows:

(7) $$d_{ij}^{ST}=\sqrt{\lambda \left[{\left(u_{i}-u_{j}\right)}^{2}+{\left(v_{i}-v_{j}\right)}^{2}\right]+\mu {\left(t_{i}-t_{j}\right)}^{2}}$$

(8) $$w_{ij}^{ST}=\exp\left\{-\left(\frac{\sqrt{\lambda \left[{\left(u_{i}-u_{j}\right)}^{2}+{\left(v_{i}-v_{j}\right)}^{2}\right]+\mu {\left(t_{i}-t_{j}\right)}^{2}}}{b_{ST}^{2}}\right)\right\}$$

where i and j are different sample areas, u and v are the coordinates of the sample area, and t is the time. The parameters λ and μ are scale factors that measure the different effects of space and time distance in the uncorrelated measurement system, and $b_{ST}$ is the bandwidth of the space-time weight function.

## 3. Results

### 3.1. Analysis of Temporal and Spatial Distribution Characteristics

**Figure 2:**
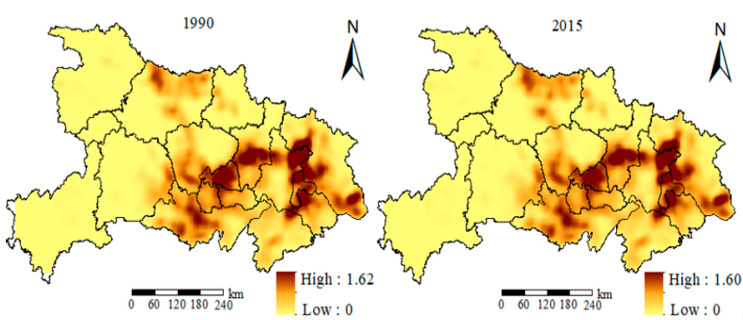
Distribution of kernel density of rural settlements from 1990 to 2015.

The method of kernel density estimation was used to produce patch distribution density maps of rural residential areas in Hubei Province in 1990 and 2015, as shown in Figure 2. In Kernel density estimation, the search radius is an important parameter [8]. After many experiments, this paper chose the search radius to be 2.5 km, which could make the smoothing effect of the nuclear density distribution map of rural settlements optimal. It can be seen from the figure that the highest nuclear density in 1990 was 1.62 cells/km^2^, and in 2010 it was 1.60 cells/km^2^, indicating that the number of plates in rural residential areas in certain areas had declined slightly. From the perspective of spatial distribution, the two-year nuclear density map had a relatively similar distribution pattern, showing spatial characteristics of dense in the southern and central areas and sparse in the west and north. It was centered on high kernel density areas in cities such as Tianmen, Ezhou, Huangshi, and Huanggang near the “Wuhan City Circle”. The spatial distribution characteristics of these high kernel density areas were related to the topography and economic development of these areas.

**Figure 3:**
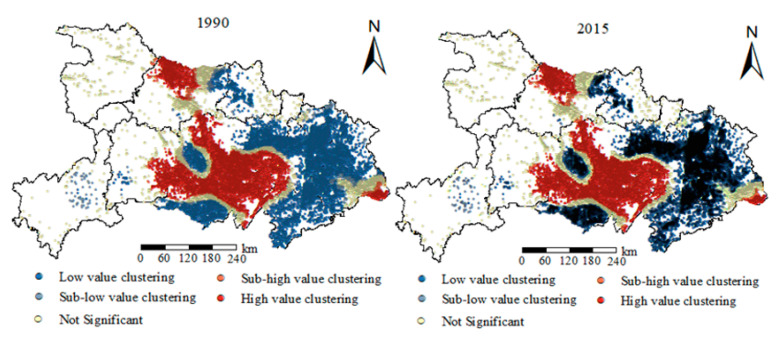
Distribution of “hot spots” of rural settlements from 1990 to 2015.

The spatial hot spots analysis tool was used to analyze the agglomeration characteristics of rural residential areas, as shown in Figure 3. The analysis results showed that from 1990 to 2015, the patch scale of rural residential areas in Hubei Province had obvious regional differentiation characteristics, showing a wave-like distribution structure of “high-low- high-low-high” from the southeast to northwest. The agglomeration characteristics of “cold and hot spots” areas continued to strengthen with time. The “hot spots” of rural residential areas were mainly distributed in Xiangyang, Yichang, and Wuhan, where the level of social and economic development was relatively high. The “cold spots” were mainly concentrated in Xiaogan, Huangshi, Huanggang, Ezhou, and Xianning. These areas are important cities in the Wuhan urban circle, which are greatly affected by the radiation of Wuhan, and a large number of farmers go to Wuhan for employment. At the same time, from 1990 to 2015, the “sub cold spots” and “sub hot spots” of rural residential areas in Hubei Province changed to “cold spots” and “hot spots” areas, and the phenomenon of gathering “cold spots” and “hot spots” further strengthened. This showed that with the development of the social economy, the agglomeration effect of rural residential areas became more and more obvious, which is mainly manifested in the urban distribution of developed economic conditions, high population concentration, good industrial development, perfect infrastructure construction, and more employment opportunities, which was more obvious in Wuhan Han region. It further revealed that the driving effect of social and economic development on rural settlements is gradually strengthening.

#### Expansion Intensity Characteristics of Rural Settlements

We calculated the land expansion intensity index of each rural residential area, and the results are shown in Table 1. From 1990 to 2015, the land expansion intensity index of rural residential areas in Hubei Province showed an overall trend of “rise-decline-rise-decline” (inverted “W” trend). In terms of a single city, the largest expansion intensity was Enshi (2.240) during the period from 2005 to 2010 and Yichang (−0.182) during the period from 1995 to 2000. From 2005 to 2015, except for Xiangyang, Suizhou, and Enshi, the index was negative, and other cities were positive. During this period, affected by the “household contract responsibility system” policy, farmers changed from simple laborers to both producers and operators in the collective economy, which greatly mobilized the enthusiasm of farmers for production and better exerted the potential of labor and land. The rural economy was obviously recovering, and farmers’ demand for residential construction land was consequently increasing. From 1995 to 2000, the index in 9 cities showed a negative value, and the absolute value of the index decreased slightly compared with the previous period. From 2000 to 2005, the expansion intensity index of rural residential land was all positive, and the index showed a relatively large increase. In 2000, the Chinese government issued a notice on the pilot work of rural tax and fee reform to explore the establishment of a standardized rural tax and fee system, which fundamentally reduced the burden on farmers. The enthusiasm of farmers engaged in agricultural production was greatly improved, and the demand for rural residential construction land increased. From 2005 to 2015, the index decreased to varying degrees compared with the previous period, and the index of most cities showed a negative value. This stage was an important period of accelerating the development of urbanization in China. The urban land areas were expanding continuously, occupying the space of rural residential construction and development to a certain extent. At the same time, due to the improvement of agricultural mechanization levels, a large number of the rural surplus labor force poured into the city to work and start a business, which also restricted the expansion of rural residential land. In addition, China’s land planning system continued to improve, rural planning, especially the new more esthetically pleasing rural construction, strengthened the consolidation and planning of rural residential areas, and paid more attention to the improvement of rural villages’ appearance and housing quality, which, to a certain extent, inhibited the extensive expansion of rural settlements.

### 3.2. Analysis of Influencing Factors

#### 3.2.1. Selection of Factors

The evolution of rural settlements is a long-term and complex process, affected by multiple factors. On the whole, it can be summarized into two categories: natural factors and social factors. In a short period of time, the natural factors are only the basic conditions for the evolution of rural settlements, the social-economic activities have greater impacts on it [43]. However, socioeconomic factors include many elements, such as economic activities and human behavior. This article referred to the research results of related scholars [44,45], combined with the availability of data, we separated farmers’ production conditions and agricultural activities from economic and social factors, thereby constructing the primary influence of the evolution of rural settlement factors. The description of each variable is shown in Table 2.

#### 3.2.2. Identification of Impact Factors Based on Geographic Detectors

Due to a large number of primary selection factors, the problem of multicollinearity is prone to occur when interpreting dependent variables, and it is impossible to estimate the explanatory effect of independent variables on dependent variables. Therefore, a geographic detector was introduced to identify invalid factors and dominant factors through screening of primary factors. On the one hand, several factors with a high degree of explanation were incorporated into the model. On the other hand, it could also achieve effective dimensionality reduction and avoid possible multicollinearity problems to the greatest extent [55]. The detection results are shown in Table 3. Based on the detection results, combined with the factor detection value and significance test, it could effectively identify invalid factors and dominant factors. Table 3 shows that the value of the four impact factors of per capita net income of farmers (PCNIF), fertilizer usage (FU), large livestock inventory (LLI), and afforestation area (AA) was small, and the value did not pass 0.05, indicating that the factor had a weak explanatory effect on the dependent variable, so these four factors were excluded. In the remaining 7 factors, the values were all greater than 0.20, indicating that these variables had a strong explanatory effect on the changes in the area of rural residential areas, and the values all passed the 0.05 significance test.

In addition, in the selection of influencing factors, the agricultural output value (AOV) and total grain output (TGO) were both representative factors in rural crop yields. In order to reduce the dimensionality of the impact factor and avoid the repeatability and invalidity of the research, the agricultural output value (AOV) and total grain output (TGO) were combined into one impact factor in the subsequent GTWR model analysis. Based on the detection results of the geographic detector, we selected the agricultural output value (AOV) with a stronger explanatory effect as a typical variable. Therefore, this paper finally selected 6 socio-economic factors that mainly affected the evolution of rural settlements, namely total rural population (TP), the rural labor force (RLF), cultivated land (CL), the agricultural output value (AOV), the total power of agricultural machinery (TPAM), and rural electricity consumption (REC).

#### 3.2.3. Temporal and Spatial Heterogeneity of Coefficients

In order to avoid the influence of collinearity, we used the geographic detector analysis method to test the collinearity of 11 factors and excluded the factors with a small q value and a p-value that failed 0.05. Finally, six socio-economic factors that mainly affected the temporal and spatial evolution of rural settlements were selected, which showed that there was no collinearity among the selected factors. According to the “Five-Year Plan” implemented by the Chinese government and the time nodes of the policies that had a greater impact on China’s rural economic and social development, this study selected 1990, 1995, 2000, 2005, 2010, and 2015 at 5-year intervals. The parameter results of the GTWR model regression analysis of the socio-economic data are shown in Table 4. Specifically, the adjusted R^2^ of the GTWR model was 0.749, indicating that the model could explain 74.90% of the rural residential area changes. Therefore, the GTWR model could effectively explore the temporal and spatial heterogeneity of the correlation between the temporal and spatial evolution of rural settlements and social-economic factors.

**Figure 4:**
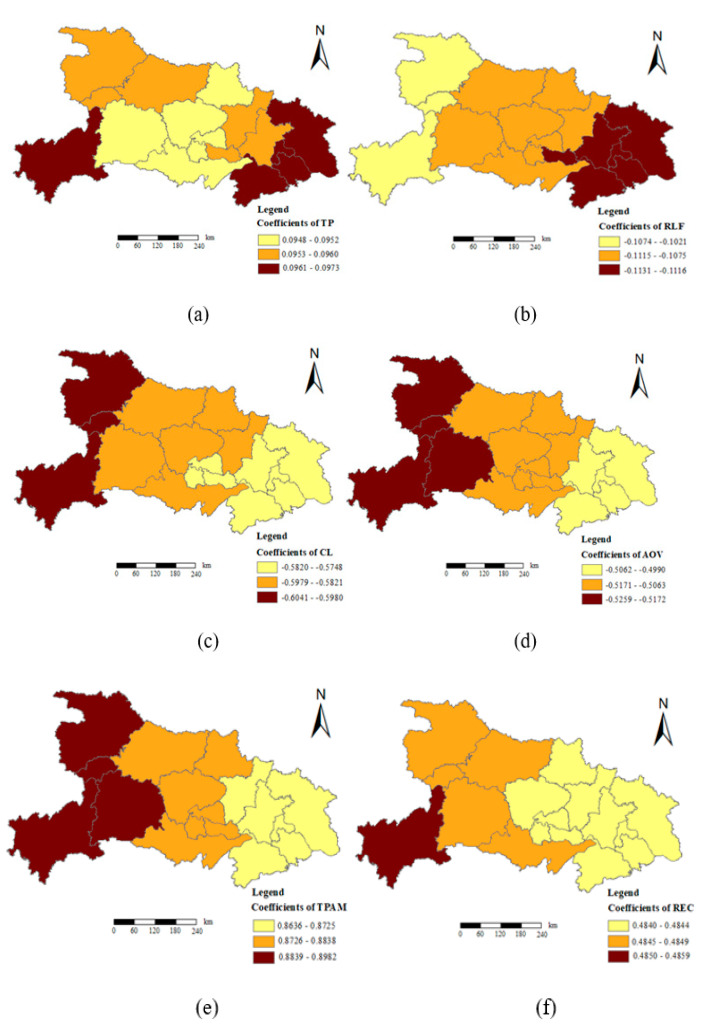
Results of GTWR of driving factors. Notes: (**a**) coefficients of Total rural population, (**b**) coefficients of Rural labor force, (**c**) coefficients of cultivated land, (**d**) coefficients of Agricultural output value, (**e**) coefficients of Total power of agricultural machinery, and (**f**) coefficients of Rural electricity consumption.

The coefficients of the variables of the GTWR model are shown in Table 5. The total rural population, the total power of agricultural machinery, and the rural electricity consumption had positive impacts on the expansion of rural residential areas, and the total power of agricultural machinery had the largest impacts. The rural labor force, the area of arable land, and the total agricultural output value had negative impacts on the expansion of the rural residential area, and the area of arable land had the most obvious impact. In order to study more clearly the spatial-temporal heterogeneity of the relationship between the six socio-economic factors and the change of rural residential area, we used the natural break-point method in arcgis10.2 to divide the coefficients with high similarity into three types (high-value area, median-value area, and low-value area) based on the GTWR estimation coefficient. The temporal and spatial distribution of each coefficient is shown in Figure 4. It revealed the temporal and spatial differences of the impacts of each variable on rural settlements.

As shown in Figure 4a, the areas with high coefficients for the total rural population were mainly concentrated in the southwest and east of Hubei Province, with Enshi, Huanggang, Huangshi, Ezhou, and Xianning being the most prominent. The median area was mainly concentrated in the northeast and central regions of Hubei Province, including Wuhan City, Xiaogan City, Xiantao City, Xiangyang City, Shiyan City, and Shenlongjia Forest Area. Low-value areas were mainly distributed in Suizhou, Jingmen, Yichang, Tianmen, Qianjiang, and Jingzhou in the southern and northern regions of Hubei Province. The average value of the regression coefficient of the total rural population was 0.096, and the standard deviation was 0.001, indicating that for every 10,000 increase in the total rural population, the area of rural residential areas in Hubei Province would increase by 0.096 hectares on average, and the degree of influence varied significantly among regions. It further illustrated that the rural population was a key factor affecting the changes of rural settlements. The increase in rural population will inevitably lead to an increase in housing demand, and housing construction will inevitably lead to the expansion of rural settlements [56,57], which is basically consistent with the research conclusions of relevant scholars. These cities in the southwest and east of Hubei Province either have relatively low levels of economic development or are located in mountainous areas with inconvenient transportation. Therefore, the total rural population has more obvious impacts on these areas.

As shown in Figure 4b, the influence of rural labor on the expansion of rural settlements was negatively correlated, and the degree of influence varied significantly across regions, showing a gradual increase from west to east. From the perspective of the absolute value of the rural labor force coefficient, the high-value areas were mainly distributed in the west of Huanggang, Huangshi, Xianning, Wuhan, Ezhou, and Xiantao, and most of these cities are close to the economically developed Wuhan with convenient transportation. A large number of rural laborers in cities in the “Wuhan Urban Circle” choose to go to Wuhan for employment or work. Some of them will choose to settle down in Wuhan, and the area of rural settlements will decrease, which is consistent with the actual situation. The median area was distributed in Xiangyang, Suizhou, Xiaogan, Yichang, Jingmen, Tianmen, Qianjiang, and Jingzhou. The low-value areas were mainly concentrated in the western mountainous areas, including Shiyan, Shenlongjia, and Enshi. The regression coefficient of rural labor was between −0.102 and −0.113, the average was −0.109, and the standard deviation was 0.003, indicating that during the study period, the impact of rural labor on the change in the area of rural residential land in Hubei Province was from 0.102 to 0.113 hectares. For every 10,000 increase in the labor force, the area of rural settlements would decrease by 0.109 hectares on average. China’s urbanization model usually shows population mobility. Located deep inland, Hubei Province is one of the main provinces with a large concentration of rural population in China. In recent years, with the continuous acceleration of the urbanization process, a large number of rural labor force mainly engaged in agriculture have gradually turned to non-agricultural labor, and the rural labor force has continued to decrease. Therefore, these cities with a larger number of agricultural laborers may have slower economic development and relatively lower income levels of farmers, which restricts the growth of rural residential land use.

As shown in Figure 4c, the areas of arable lands had negative impacts on the expansion of rural settlements, and the degree of impact decreased from west to east. The high-value areas of influence were mainly distributed in Shiyan, Shenlongjia, and Enshi in the west. Most of these cities are located in the mountains of western Hubei, with complex topography, small arable land, a large proportion of forest land, inconvenient transportation, and relatively lagging social and economic development. The median area was mainly concentrated in Xiangyang, Yichang, Suizhou, Jingmen, and Jingzhou in the middle. The low-value areas were mainly concentrated in Tianmen, Qianjiang, and Xiantao in the central region, and Huanggang, Wuhan, Ezhou, Huangshi, and Xianning in the eastern regions. The central and southern regions of Hubei Province are composed of plains and hills, which are mainly concentrated areas of arable land. The regression coefficient of the cultivated land area was between −0.604 and −0.575, the average value of the coefficient was −0.584, and the standard deviation was 0.009, indicating that the impact of cultivated land area on rural residential area changes during this period was between 0.575 and 0.604 hectares. If the area of arable land in this area increased by 666.667 hectares, the average land area of rural residential areas would decrease to 0.584 hectares. The research time of this article was from 1990 to 2015, and this period spans a long time. In 1978, China’s “Household Responsibility System for Production-related Contracting” was first established in Xiaogang Village, and then it has been continuously promoted in rural China. In 2000, the Chinese government issued the “notice on the pilot work of rural tax and fee reform”, which explored the establishment of a standardized rural tax and fee system and fundamentally reduced the burden of farmers. Cultivated land has gradually become an important factor affecting China’s economic development. At the same time, the enthusiasm of farmers engaged in agricultural activities and the demand for housing construction have also increased. During this period, the impact of cultivated land on rural residential land was positive. However, since the beginning of the 20th century, China has vigorously promoted the development of agricultural modernization under the guidance of the comprehensive, coordinated, and sustainable development concept. China has carried out the strategy of rural industrialization and rural urbanization, and the government has also issued a large number of policies of “Financing Agriculture” and “Benefiting Agriculture”, which have significantly improved the level of agricultural modernization. During this period, the level of agricultural mechanization in China was greatly improved, especially the industrialization and large-scale operation of agriculture directly or indirectly liberated a large number of rural laborers. The increase in the area of arable land provides conditions for agricultural industrialization and large-scale operations. A large number of rural surplus laborers go to cities to work or start businesses, and the need for rural housing construction is reduced, which then restricts the increase in the area of rural residential land. Therefore, from the perspective of the entire study period, the increase in farmland area has restricted the expansion of rural residential land to a certain extent.

As shown in Figure 4d, the total agricultural output value had negative impacts on the expansion of rural settlements, and the degree of impact varied significantly between regions. From the perspective of the absolute values of the coefficients, its impact on the changes in the area of rural residential areas showed a decreasing trend from west to east. The high-value areas of the coefficients were distributed in Shiyan, Shenlongjia, Yichang, and Enshi in the west. The median area was concentrated in Xiangyang and Suizhou in the north, Jingmen, Xiaogan, Tianmen, Qianjiang, and Xiantao in the middle, and Jingzhou in the south. The low-value areas were mainly distributed in the east of Huanggang, Wuhan, Ezhou, Huangshi, and Xianning. The range of the agricultural output value coefficient was −0.526 to −0.499, the average value was −0.510, and the standard deviation was 0.008, indicating that the higher the agricultural output value, the smaller the impact on the change of rural residential land area in the study area. The change in the area of rural residential areas caused by an increase of 1 million yuan in total agricultural output value ranged from 0.499 to 0.526 hectares, and the area of rural residential areas decreased by 0.510 hectares on average. The increase in total agricultural output value was conducive to the improvement of farmers’ income level and the increase in farmers’ net income may have positive and negative effects on the growth of rural residential lands. While the increase in farmers’ income provided conditions for farmers to increase the area of housing and stimulate them to build new houses to improve their living conditions, on the other hand, a large number of farmers may also buy houses in cities and towns, which may cause a large-scale rural population to migrate to cities. Therefore, the increase of farmers’ income and the change of consciousness had considerable impacts on the land use space of rural residential areas, which may have inhibited the growth of rural residential land use [58]. This result was more consistent with the conclusions in related studies.

As shown in Figure 4e, the total power of agricultural machinery had positive effects on the expansion of rural settlements, and the degree of influence decreased from west to east. The high-value area, medium-value area, and low-value area of its coefficient distribution were similar to the distribution pattern of total agricultural output value mentioned above, except that Xiaogan was distributed in the low-value areas. The coefficient of the total power of agricultural machinery ranged from 0.864 to 0.898, with an average value of 0.878, indicating that the greater the total power of agricultural machinery, the greater the impact on the changes in the area of rural residential land. The total power of agricultural machinery increased by 10,000 kilowatts, and the possible impacts of the change in the area of rural residential lands were 0.864 to 0.898 hectares, which would promote an average increase of 0.878 hectares of construction land in rural residential areas. This factor was also an important indicator reflecting the level of regional agricultural modernization. Generally, the greater the total power of agricultural machinery, the higher the degree of intensification of agricultural production in the region. The improvement of the total power of agricultural machinery will greatly stimulate the enthusiasm of farmers to engage in agricultural production, and transform agricultural production activities from the traditional labels of “hard work” and “low income” to “mechanization” and specialization. In the long run, it has effectively stimulated migrant workers who originally entered the cities to return to the countryside to engage in agricultural production activities, which also directly promoted the increase in land demand for rural residential areas.

As shown in Figure 4f, rural electricity consumption had positive impacts on the expansion of rural residential areas and had obvious spatial heterogeneity characteristics. Electricity consumption was a key indicator for measuring rural economic development, and it reflected the scale of agricultural production and the living standards of rural residents to a certain extent. The high-value areas of rural electricity consumption coefficient were distributed in Enshi, mainly because Enshi is located in the western Hubei mountain area. The distribution of rural residential areas is relatively scattered, economic development may be relatively slow, rural infrastructure facilities are relatively lagging, and the quality of life of farmers is not high. Therefore, electricity consumption had significant impacts on the changes in the area of rural residential areas in the region. The median area was mainly distributed in Shiyan, Shenlongjia forest area, Xiangyang, Yichang, and Jingzhou. The low-value areas were mainly distributed in Suizhou, Jingmen, Tianmen, Qianjiang, Xiantao, Xiaogan, Wuhan, Huanggang, Ezhou, Huangshi, and Xianning. As a national central city, Wuhan has a developed economy, complete infrastructure, and convenient transportation. Cities in low-value areas are close to Wuhan, and most cities are an important part of the “Wuhan City Circle”. The cities are driven by the radiation of Wuhan and have relatively high levels of economic development. Therefore, rural electricity consumption had a relatively small impact on the changes in the area of rural residential areas in these regions. Specifically, the average value of the rural electricity consumption coefficient was 0.484, which meant that every increase in rural electricity consumption in the study area by 10,000 kilowatts would increase the area of rural residential areas by an average of 0.484 hectares. Rural electricity consumption was an important manifestation of the level of rural economic development and the quality of life of farmers. It had a positive effect on the increase of rural residential land use, indicating that the rural economically developed areas had higher land production efficiency. These rural areas paid more attention to the intensive and economical land use of residential areas, and farmers had higher pursuits of housing quality and living standards, which stimulated farmers’ demand for residential construction land to a certain extent.

## 4. Conclusions

After the implementation of economic reforms in 1978, China’s rural areas have experienced unprecedented development, and the pattern of construction land for rural settlements has also undergone great changes. In the context of China’s current “village revitalization strategy”, to realize the overall revitalization of the countryside and fully realize the goals of strong agriculture, beautiful countryside, and rich farmers, it is necessary to rationally plan rural settlements and clarify the relationship between rural residents and socio-economic factors. Therefore, in order to investigate the relationship between the socio-economic driving factors and the land use pattern of rural residential areas, we first analyzed the kernel density, patterns of cold spots and hot spots, and expansion intensity characteristics of rural residential land use. Then, combined with the geographic detector method, six socio-economic factors that affect the land-use changes of rural residential areas were selected, and the GTWR model was used to explore the relationship between the six socio-economic factors and the changes in the rural residential areas.

The study found that the kernel density of rural settlements decreased from 1.62 villages/km^2^ in 1990 to 1.60 villages/km^2^ in 2015. Moreover, from 1990 to 2015, the patch scale of rural residential areas has obvious regional differentiation characteristics, showing a wave-like distribution structure of “high-low high-low-high” from southeast to northwest. As time progresses, the agglomeration characteristics of “cold and hot spots” in rural residential areas have shown a growing trend. It is strongly associated with the level of urban social and economic development in Hubei Province. The agglomeration effect of rural residential areas in the “Wuhan Urban Circle” area is more obvious, indicating that the driving effect of social and economic development on rural residential areas is gradually strengthening.

Secondly, during the study period, the rural land expansion intensity index showed an inverted “W” trend (rising-falling-rising-falling). From 1990 to 1995, except for Xiangyang, Suizhou, and Enshi, the expansion intensity index of other cities is positive. From 1995 to 2000, the rural land expansion intensity index in 9 cities in Hubei Province is negative. Compared with the previous period, the absolute value of the expansion intensity index decreased slightly.

Finally, based on the geographic detector analysis method and the GTWR model, we found that the total rural population, the total power of agricultural machinery, and rural electricity consumption have promoted the expansion of rural settlements, the regression coefficients were 0.096, 0.484, and 0.878, respectively. Cultivated land, agricultural output value, and rural labor force have negative impacts on the expansion, the regression coefficients of the village were −0.584, −0.510, and −0.109, respectively. Scholars such as Feng Changchun believe that among the social and economic factors in China’s rural areas, the increase in rural permanent residents, the increase in rural residents’ per capita net income and per capita agricultural production efficiency, and the reduction in the size of rural households will all promote the expansion of rural settlements [59]. Yang Yong believes that the increase in the total population and the number of households in the region will promote the growth of rural residential land-use [60]. This is basically consistent with this conclusion and also in line with the reality of the evolution and development of rural settlements in China.

## 5. Discussion

This study uses various methods to research the spatial-temporal characteristics and the driving factors of rural settlements in the low hilly regions, it has some reference value for the government to adopt relevant urban and rural sustainable strategies. However, the research is still in the preliminary exploration stage, and it still has some shortcomings. For example, due to the limitation of data, this paper only analyzes the construction land of rural residential areas in 17 cities in Hubei Province. Moreover, it has not explored rural settlements at the county and township scales, but research on small-scale rural settlements is also an important direction of current research. Then, this paper did not find the factors that lead to small-scale changes in rural residential land use (educational land, administrative and public service land, commercial land, and industrial land). In addition, more socio-economic factors, such as traffic factors, policy factors, and point of interest (POI) factors, should also be considered in the research on the evolution of rural settlements. Therefore, we still need to strengthen the exploration of these aspects in future research.

## Figures and Tables

**Table 1:** Expansion Intensity Index of Rural Settlements in Hubei Province.

City	1990~1995	1995~2000	2000~2005	2005~2010	2010~2015
Wuhan	0.147	0.070	0.191	−0.076	−0.045
Shiyan	0.163	−0.043	0.271	−0.295	0.062
Xiangyang	−0.015	0.092	0.259	1.065	0.028
Suizhou	−0.024	0.121	0.231	0.645	0.251
Jingmen	0.124	−0.117	0.160	0.152	−0.032
Xiaogan	0.093	0.017	0.106	−0.028	−0.003
Yichang	0.248	−0.182	0.164	0.075	−0.008
Huangguang	0.121	−0.074	0.104	−0.055	−0.044
Ezhou	0.199	−0.179	0.114	−0.153	−0.011
Jingzhou	0.094	−0.048	0.075	0.075	−0.012
Huangshi	0.227	−0.150	0.119	0.110	−0.056
Xianning	0.038	0.049	0.054	−0.066	−0.015
Enshi	−0.105	0.270	1.104	2.240	0.319
Shenlongjia	0.696	−0.107	0.117	−0.350	−0.013
Tianmen	0.028	0.062	0.078	−0.151	−0.023
Qianjiang	0.001	0.012	0.051	0.054	−0.019
Xiantao	0.051	−0.010	0.006	0.020	0.024

**Table 2:** Description of primary variables.

Variables	Units	Variable Descriptions	References
Total rural population (TP)	Ten thousand people	Statistics of the total rural population at the end of each city.	[46]
Per capita net income of farmers (PCNIF)	Yuan	The total income of rural households from various sources in the current year is the sum of income after deducting the expenses incurred accordingly.	[37]
Rural labor force (RLF)	Ten thousand people	The number of rural populations within working age who have the ability to work and often participate in social labor.	[46,47]
Cultivated land (CL)	666.667 hectares	It can be used to grow crops and often plow and hoe fields.	[37,46,48]
Agricultural output value (AOV)	Ten thousand yuan	The total amount of all agricultural, forestry, animal husbandry, and fishery products expressed in currency in a certain period (usually a year).	[49]
Total power of agricultural machinery (TPAM)	kilowatt	Mainly used for the power of various power machinery in agriculture, forestry, animal husbandry, and fishery.	[34,50]
Rural electricity consumption (REC)	Ten thousand kilowatt hours	During the current year, the total annual electricity consumption for rural production and living after deducting the electricity consumption of state-owned economic units in the countryside.	[50]
Fertilizer usage (FU)	Ton	The amounts of chemical fertilizers actually used in agricultural production this year.	[51]
Large livestock inventory (LLI)	Ten thousand	The amounts of livestock raised in a certain period of time (a year).	[50,52]
Total grain output (TGO)	Ten thousand tons	The total amount of grain produced by agricultural producers and operators in a calendar year.	[53]
Afforestation area (AA)	hm^2^	The sum of the area of artificial afforestation on barren hills and wasteland and the afforestation area of plane sowing.	[54]

**Table 3:** Geographic detector detection results.

Detection Factors	TP	PCNIF	RLF	CL	AOV	TPAM	REC	FU	LLI	TGO	AA
q	0.403	0.055	0.278	0.308	0.310	0.346	0.393	0.171	0.119	0.206	0.059
p	0.000	0.358	0.000	0.000	0.000	0.000	0.000	0.061	0.051	0.000	0.301

**Table 4:** Geographically temporally weighted regression model (GTWR) model indicators.

Statistics	Parameters
Bandwidth	1.983
Residual Squares	75.561
Sigma	0.861
AICc	280.522
R^2^	0.749
Adjusted R^2^	0.730
Spatiotemporal Distance Ratio (Based on Automatic Optimization)	0.269

**Table 5:** Statistical description of GTWR coefficients.

Variables	Mean	SD	Min	Median	Max
TP	0.096	0.001	0.095	0.095	0.097
RLF	−0.109	0.003	−0.113	−0.110	−0.102
CL	−0.584	0.009	−0.604	−0.582	−0.575
AOV	−0.510	0.008	−0.526	−0.509	−0.499
TPAM	0.878	0.011	0.864	0.877	0.898
REC	0.484	0.000	0.484	0.484	0.486

## Data Availability

Data available on request. The detailed experimental data presented in this study are available on request from the corresponding author.
